# NADPH oxidase mediates β-amyloid peptide-induced activation of ERK in hippocampal organotypic cultures

**DOI:** 10.1186/1756-6606-2-31

**Published:** 2009-10-05

**Authors:** Faridis Serrano, Angela Chang, Caterina Hernandez, Robia G Pautler, J David Sweatt, Eric Klann

**Affiliations:** 1 Department of Molecular Physiology & Biophysics, Baylor College of Medicine, Houston, TX, USA; 2 Department of Neuroscience, Baylor College of Medicine, Houston, TX, USA; 3 Department of Neurobiology, University of Alabama, Birmingham, AL, USA; 4 Center for Neural Science, New York University, New York, NY, USA

## Abstract

**Background:**

Previous studies have shown that beta amyloid (Aβ) peptide triggers the activation of several signal transduction cascades in the hippocampus, including the extracellular signal-regulated kinase (ERK) cascade. In this study we sought to characterize the cellular localization of phosphorylated, active ERK in organotypic hippocampal cultures after acute exposure to either Aβ (1-42) or nicotine.

**Results:**

We observed that Aβ and nicotine increased the levels of active ERK in distinct cellular localizations. We also examined whether phospho-ERK was regulated by redox signaling mechanisms and found that increases in active ERK induced by Aβ and nicotine were blocked by inhibitors of NADPH oxidase.

**Conclusion:**

Our findings indicate that NADPH oxidase-dependent redox signaling is required for Aβ-induced activation of ERK, and suggest a similar mechanism may occur during early stages of Alzheimer's disease.

## Background

Beta amyloid (Aβ) peptide is typically considered neurotoxic as it is present in high levels and accumulates in plaques in the brain of Alzheimer's disease (AD) patients [1]. However, Aβ also is present in the normal brain at lower levels than in the disease state, suggesting that it has normal cellular and physiological functions [2]. Therefore, studies of the impact of exposure of the brain to Aβ could provide important information with respect to its role during both physiological and pathophysiological processes. It has been demonstrated that acute treatment of organotypic hippocampal cultures with nanomolar concentrations of oligomeric Aβ (1-42) leads to the activation of extracellular signal-regulated kinase (ERK) via the alpha 7 nicotinic acetylcholine receptor (α 7nAChR) [3,4]. This signaling cascade involves an increase in calcium and activation of ERK mediated by phosphoinositide-3 kinase (PI3K), which is followed by increases in CREB phosphorylation [3-6]. Similar to Aβ, nicotine treatment causes ERK activation via α 7nAChR and requires PI3K. However, protein kinase A (PKA) appears to be an upstream regulator of ERK activation after nicotine treatment, but not after Aβ treatment [3]. The activation of different signaling cascades by Aβ and nicotine suggest the possibility of differential cellular activation and/or localization by these molecules. In this study we sought to characterize the cellular localization of the Aβ- and nicotine-induced increases in active ERK in area CA1 of hippocampal organotypic cultures.

Reactive oxygen species (ROS) typically are characterized as molecules involved in neurotoxicity and neurodegeneration [7]. However, substantial evidence suggests that ROS also function as small messenger molecules that are normal components of signal transduction cascades required for physiological processes such as synaptic plasticity, learning, and memory [8]. Because it is known that Aβ peptides can produce ROS, it is important to identify the source of the ROS production, as well as downstream effectors of ROS. NADPH oxidase is particularly intriguing as a possible source of ROS produced by Aβ peptides. NADPH oxidase is a tightly regulated, multiprotein enzyme that produces large quantities of the ROS superoxide that has been well characterized in phagocytic cells [9]. The active oxidase is made up of several protein components, including two membrane proteins, gp91^*phox *^and p22^*phox *^(also known as cytochrome *b*_558_), and three cytosolic proteins, p47^*phox*^, p67^*phox*^, and the small GTP-binding protein Rac. Upon stimulation, the cytosolic proteins translocate to the membrane to form a complex with cytochrome *b*_558_, which results in enzymatic activation and production of superoxide [10,11]. In recent years, NADPH oxidase and NADPH oxidase-like enzymes (NOX) have been described in non-phagocytic cells [12,13]. For example, NADPH oxidase has been observed in peripheral neurons [14,15], cerebral cortical neurons [16,17], and cerebellar Purkinje neurons [18] among others. NADPH oxidase also is present in the hippocampus, including pyramidal neurons in area CA1 [19,20]. Superoxide produced by NADPH oxidase predominately has been studied with respect to neurotoxicity [15,21], but there also is evidence that superoxide is required for hippocampal long-term potentiation and hippocampus-dependent memory [22-25]. Therefore, NADPH oxidase could play an important role not only in disease conditions, but also during normal neuronal function. Interestingly, it has been demonstrated that in brains of AD patients there is an increase in the expression of NADPH oxidase proteins [26] and that AD model mice lacking gp91^*phox *^do not develop oxidative stress, cerebrovascular dysfunction, or behavioral deficits [27]. Furthermore, *in vitro *studies using cortical neurons have demonstrated an involvement of NADPH oxidase in response to Aβ (1-42) stimulation [28]. These observations suggest that NADPH oxidase could contribute to the oxidative stress associated with AD, and also implicate it in regulating signal transduction cascades under more physiological conditions. In this study we sought to investigate whether Aβ peptide regulates the ERK signaling cascade via NADPH oxidase in hippocampal organotypic cultures.

## Results

### Differential cellular expression of ERK after Aβ- and nicotine-treatments

**Figure 1:**
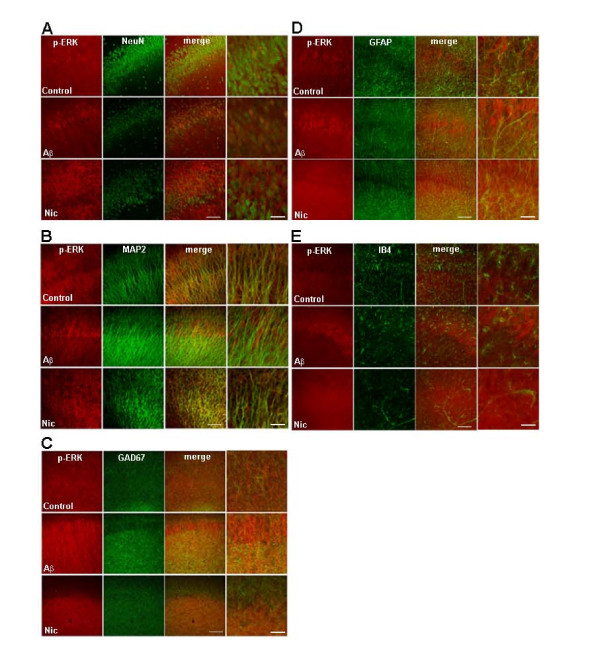
**Aβ- and nicotine-induced activation of ERK in cultured hippocampal slices**. Hippocampal slice cultures were treated with culture media, media containing Aβ (100 nM), or media containing nicotine (500 nM) for either 5 min or 10 min, respectively. Confocal images of hippocampal area CA1 were obtained from slices that were double labeled using antibodies specific for phospho-ERK (red) and A) the neuronal marker NeuN (green), B) the dendritic marker MAP2 (green), C) a marker for GABAergic interneurons GAD67 (green), D) the astrocytic marker GFAP (green), and E) the microglial marker IB4 (green). Dual labeling is indicated by yellow/orange. All the images were taken with a 20× objective and inserts represent higher magnifications (optical zoom 3.0). The scale bars are the same in all images and represent 20 μm and 10 μm, respectively.

It has been demonstrated that both Aβ peptide and nicotine induce the activation of ERK in organotypic hippocampal cultures [4]. To better understand Aβ- and nicotine-induced activation of ERK, we determined the cellular localization of the active form of ERK (phospho-ERK) after each treatment. Using antibodies against several cell markers we observed differences in the cellular localization of active ERK immunoreactivity in Aβ- and nicotine-treated organotypic cultures (Figure 1). Treatment with Aβ resulted in the activation of ERK in both neuronal cell bodies and dendrites, as phospho-ERK immunoreactivity was present in stratum pyramidale and stratum radiatum of hippocampal area CA1 (Figure 1A and 1B). In contrast, treatment of the organotypic cultures with nicotine did not appear to activate ERK in the cell bodies of the pyramidal neurons, but rather in stratum radiatum (Figure 1A and 1B). We also treated hippocampal slices with a higher concentration of nicotine (500 μM) and found the pattern of ERK activation was similar and not localized to the cell bodies (data not shown). Because stratum radiatum includes pyramidal neuron dendrites and interneurons, we also used antibodies against GABAergic interneurons to examine whether they expressed active ERK. Aβ- and nicotine-induced increases in active ERK in stratum radiatum did not appear to co-localize with the GABAergic marker GAD67, as the staining did not show a punctuate appearance (Figure 1C). Neither Aβ nor nicotine appeared to cause activation of active ERK in either astrocytes (Figure 1D) or microglia (Figure 1E). These observations suggest that the activation of ERK after either Aβ or nicotine treatment is restricted to neurons and is not present in glial cells. Moreover, these stimuli appear to trigger activation of ERK in distinct neuronal compartments.

### Aβ peptide-induced activation of ERK is blocked by the NADPH oxidase inhibitors apocynin and DPI

**Figure 2:**
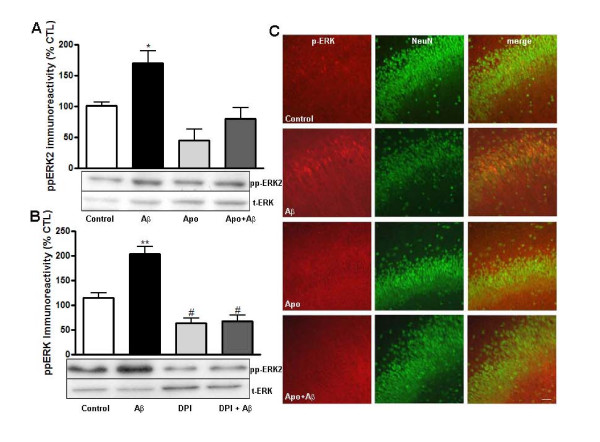
**Aβ peptide-induced activation of ERK is blocked by the NADPH oxidase inhibitors apocynin and DPI**. Hippocampal slice cultures were pretreated for either 25 or 55 min with media in the presence or absence of either 100 μM apocynin (A) or 10 μM DPI (B), followed by incubation with Aβ (100 nM, 5 min). A, B) Western blots. Phospho-ERK immunoreactivity was normalized to total ERK and expressed as percent of control. Data represent the mean ± SEM; n = 7-10; *p < 0.01; **p < 0.001; #p < 0.05. C) Immunocytochemistry. Confocal images of hippocampal area CA1 were obtained from slices that were double labeled using antibodies specific for phospho-ERK (red) and the neuronal marker NeuN (green). Dual labeling is indicated by yellow/orange. Images were taken with a 20× objective and the scale bar represents 20 μm.

In order to investigate whether the Aβ-induced activation of ERK was mediated by NADPH oxidase, we treated organotypic cultures with Aβ in the presence or absence of apocynin (100 μM) and diphenyleneiodomium (10 μM; DPI). These two compounds inhibit NADPH oxidase by blocking distinct sites: apocynin blocks the p47^*phox *^subunit and DPI blocks the gp91^*phox *^subunit. Western blot analysis indicated that treatment of hippocampal organotyopic cultures with Aβ caused an increase in ERK phosphorylation compared to vehicle treatment (Figure 2A) supporting previous observations [3,4]. Furthermore, we found that treatment with Aβ in the presence of apocynin failed to increase ERK phosphorylation above control levels (Figure 2A). Similar results were observed when hippocampal organotypic cultures were exposed to Aβ in the presence of another NADPH oxidase inhibitor, DPI (Figure 2B). Immunocytochemical analysis also demonstrated that increases in active ERK in both pyramidal neuron cell bodies and dendrites induced by Aβ treatment were blocked by apocynin (Figure 2C). These observations suggest that superoxide produced by the NADPH oxidase is necessary for the Aβ-induced neuronal activation of ERK.

### Nicotine-induced activation of ERK is blocked by the NADPH oxidase inhibitors apocynin and DPI

**Figure 3:**
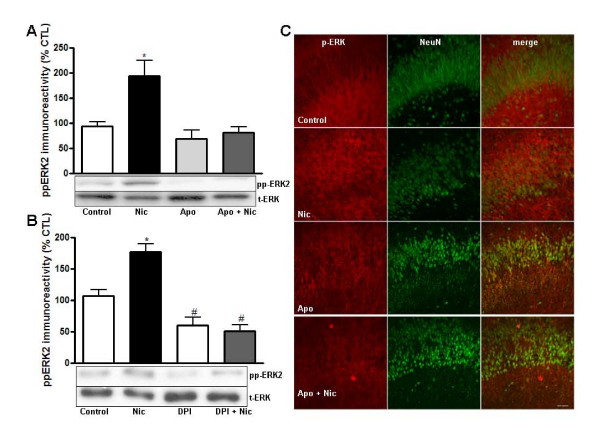
**Nicotine-induced activation of ERK is blocked by the NADPH oxidase inhibitors apocynin and DPI**. Hippocampal slice cultures were pretreated for 25 or 55 min with media in the presence or absence of either 100 μM apocynin (A) or 10 μM DPI (B), followed by incubation with nicotine (500 nM, 10 min). A, B) Western blots. Phospho-ERK immunoreactivity was normalized to total ERK and expressed as percent of control. Data represent the mean ± SEM; n = 6-8; * p < 0.01, ** p < 0.001. C) Immunocytochemistry. Confocal images of hippocampal area CA1 were obtained from slices that were double labeled using antibodies specific for phospho-ERK (red) and the neuronal marker NeuN (green). Dual labeling is indicated by yellow/orange. Images were taken with a 20× objective and the scale bar represents 20 μm.

Because Aβ peptides have been shown to interact with nicotinic receptors to trigger the activation of ERK [4], we asked whether nicotine-induced activation of ERK also requires NADPH oxidase. Western blot analysis demonstrated that treatment of hippocampal organotyopic cultures with nicotine caused an increase in phospho-ERK that was blocked by apocynin (Figure 3A). Similar results were observed when hippocampal organotypic cultures were exposed to nicotine in the presence of DPI (Figure 3B). Immunocytochemical analysis also demonstrated that increases in active ERK in the stratum radiatum area induced by nicotine treatment were blocked by apocynin (Figure 3C). These observations suggest that superoxide produced by NADPH oxidase is necessary for the nicotine-induced neuronal activation of ERK.

### Contribution of α7nAChR to the Aβ- and nicotine-induced activation of ERK

**Figure 4:**
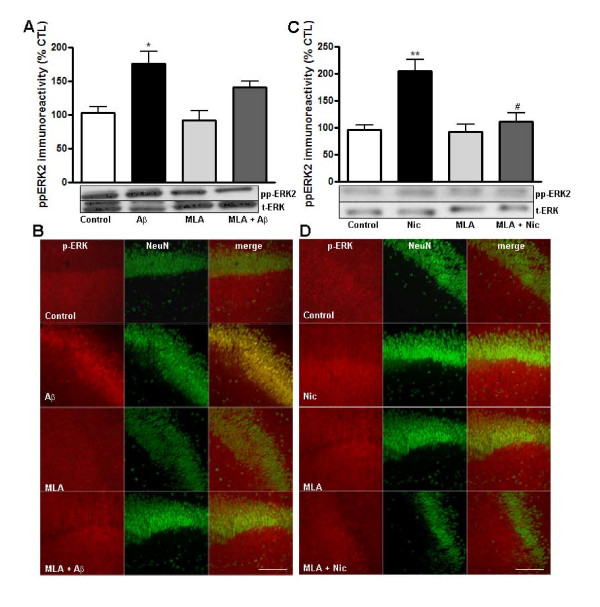
**Aβ- and nicotine-induced activation of ERK is decreased by the α7nAChR inhibitor MLA**. Hippocampal slice cultures were pretreated for 30 min with media in the presence or absence of MLA (10 nM) followed by incubation with Aβ (100 nM, 5 min). A) Western blots. Phospho-ERK immunoreactivity was normalized to total ERK and expressed as percent of control. Data represent the mean ± SEM. n = 3-6; *p < 0.01. B) Immunocytochemistry. Confocal images were obtained from slices that were double labeled using antibodies specific for phospho-ERK (red) and the neuronal marker NeuN (green). Dual labeling is indicated by yellow/orange. Hippocampal slice cultures were pretreated for 30 min with media in the presence or absence of MLA (10 nM) followed by incubation with nicotine (500 nM, 5 min). C) Western blots. Phospho-ERK immunoreactivity was normalized to total ERK and expressed as percent of control. Data represent the mean ± SEM. n = 3-6, **p < 0.001. D) Immunocytochemistry. Confocal images were obtained from slices that were double labeled using antibodies specific for phospho-ERK (red) and the neuronal marker NeuN (green). Dual labeling is indicated by yellow/orange. Images were taken with a 20× objective and the scale bar represents 50 μm.

It has been previously demonstrated that Aβ interacts with nicotinic acetylcholine receptors [3-6,29,30]. In particular, there is evidence indicating that micromolar concentrations of methyllycaconitine (MLA) blocks Aβ-induced increases in ERK phophorylation in hippocampal organotypic slices [4]. It also has been demonstrated that MLA is more selective for the α7 subunit of nAChRs at nanomolar concentrations [31,32]. Western blot analysis demonstrated that in the presence of nanomolar concentrations of MLA, Aβ failed to cause a significant increase in the levels of phospho-ERK compared to control (Figure 4A). Immunocytochemical examination confirmed that the Aβ-induced increase in phospho-ERK immunoreactivity was reduced by MLA in both the pyramidal cell bodies and dendrites (Figure 4B). Similarly, the nicotine-induced increase in active ERK was inhibited in the presence of MLA (Figure 4C). The increases in active ERK in the dendritic region induced by nicotine treatment also were blocked by MLA treatment (Figure 4D). Taken together, these findings support previous observations demonstrating a role for α7nAChR in regulating ERK activation after Aβ and nicotine treatments. Although there was not a significant increase in ERK activation when Aβ was added to slices in the presence of nanomolar concentrations of MLA, there was a trend for increased active ERK. This suggests the possibility that Aβ impacts other nicotinic receptors and signaling pathways to trigger activation of ERK.

## Discussion

Acute treatments of organotypic hippocampal cultures with either Aβ or nicotine have been found to increase ERK phosphorylation [3,4]. Herein, we have expanded on those previous observations by using immunocytochemistry and have determined that the increase in ERK phosphorylation after Aβ treatment was localized to the cell soma and dendrites of hippocampal CA1 neurons (Figure 1). Interestingly, our data indicate that nicotine treatment induced ERK phosphorylation only in the dendritic compartment because the cell soma lacked enhanced phospho-ERK immunoreactivity. The differences in the localization of phospho-ERK activation following Aβ and nicotine suggest that ERK activation is mediated by different upstream signaling modules. Consistent with this observation, it has been shown that both Aβ and nicotine trigger the activation of ERK, but by using distinct intermediate kinases [3]. More studies are necessary to specifically address the implications of this differential compartmentalization of ERK phosphorylation after these two treatments.

Importantly, we also observed that acute treatment with nanomolar concentrations of Aβ induced the activation of ERK in neurons, but not glial cells (Figure 1), as we did not observe dual staining for phospho-ERK with markers for either astrocytes or microglia. Similarly, nicotine treatment showed no evidence of active ERK immunoreactivity in glia cells (Figure 1). It previously was observed that Aβ treatment can cause increases in active ERK in glia cells; however, these increases in active ERK were after chronic exposure to Aβ, suggesting that the cells were responding to neurotoxic events [33-35]. In addition, chronic treatment with nanomolar concentrations of either monomeric or oligomeric Aβ, as well as higher concentrations (micromolar) of fibrillar Aβ, are typically associated with ERK activation leading to neurotoxicity [3,36]. Furthermore, it has been demonstrated that in early stages of AD there is a transient increase in active ERK in astrocytes that might represent a response to axonal damage and presynaptic loss [37]. All together, these findings suggest a scenario where low concentrations of Aβ induces the physiological activation of ERK in neurons, whereas higher concentrations of Aβ induces the pathophysiological activation of ERK in glial cells.

Earlier studies demonstrated that treatment of acute hippocampal slices with a superoxide-generating system causes ERK phosphorylation [38]. The involvement of ERK signaling in synaptic plasticity and memory function has been studied intensely [39] and several studies have demonstrated an important role for NADPH oxidase and ROS in synaptic plasticity and learning and memory [22,40]. Moreover, N-methyl-D-aspartate (NMDA) receptor-dependent activation of ERK in the hippocampus is mediated by NADPH oxidase [40]. Our findings strongly suggest that both Aβ and nicotine treatments trigger an increase in ERK phosphorylation in hippocampal pyramidal neurons via NADPH oxidase (Figure 2) and are in agreement with a previous study in cortical neurons [28]. There also is evidence that neutrophils stimulated with Aβ fragments (25-35) at micromolar concentrations activate ERK, resulting in the activation of NADPH oxidase to produce superoxide [41]. This apparent difference in signaling modules could be due to the cell type, Aβ peptide solution, or the Aβ concentration and time of incubation used. Regardless, there is extensive evidence that superoxide produced by NADPH oxidase triggers ERK activation during both physiological and pathophysiological conditions [42-44]. An involvement of NADPH oxidase in ERK-mediated signaling after nicotine treatment has not been studied extensively. However, it was shown that in non-neuronal cells nicotine can modulate cell function via both NADPH oxidase and ERK [45]. Taken together, our results are in agreement with others and point to a significant role for NADPH oxidase in ERK activation by either Aβ or nicotine.

In agreement with previous studies [4] we found that nanomolar concentrations of MLA inhibited the increase in active ERK after Aβ treatment (Figure 4). The importance of the interaction of Aβ with α7nAChRs during both normal synaptic plasticity and pathologic conditions such as AD is well recognized [46], but further studies are necessary to increase our understanding of the signaling cascades mediating these processes.

At the present time the subcellular localization of NADPH oxidase involved in the Aβ/ERK signaling pathway in the hippocampus is not clear. Our findings indicate that Aβ treatment causes the activation of ERK in pyramidal neuron cell bodies and dendrites and we have previously shown a synaptic localization of NADPH oxidase in the hippocampus [20]. One possible scenario is that Aβ causes the activation of NADPH oxidase postsynaptically and that the production of ROS causes the activation of ERK in both cell bodies and dendritic compartments. Another possibility is that NADPH oxidase is present presynaptically and that after Aβ treatment, the superoxide that is produced travels trans-synaptically, leading to the activation of ERK in postsynaptic pyramidal neurons. These possibilities remain to be examined.

Whether the involvement of the α7nChR in the Aβ-induced ERK activation is upstream of the NADPH oxidase activation is still unclear. α7nChRs have been shown to be present in both presynaptic and postsynaptic compartments of pyramidal neurons, as well as in GABAergic interneurons [47-49]. It has been suggested that nAChRs at presynaptic locations on excitatory neurons could impact hippocampal synaptic plasticity [49]. Thus, if the nicotinic receptor activation is indeed upstream to NADPH oxidase activation, one can speculate that Aβ binds to presynaptic nAChRs, enhancing release of glutamate, which would lead to increases in postsynaptic calcium followed by activation of NADPH oxidase and ERK. Another possibility is that Aβ binds to nicotinic receptors in the postsynaptic neuron causing depolarization, NADPH oxidase activation, and subsequent ERK activation. More studies will be necessary to directly investigate which additional mechanisms are involved in the Aβ-induced ERK activation.

## Conclusion

Our findings indicate that NADPH oxidase-dependent redox signaling is required for Aβ-induced activation of ERK in hippocampal neurons. These observations expand our understanding of the regulation and signaling cascades activated by Aβ and provide insight about the signaling cascades that might be triggered during the early stages of Alzheimer's disease.

## Methods

### Hippocampal slice cultures and treatment

Slice cultures were prepared from P7 Sprague-Dawley rat pups and maintained in culture according to the method of Stoppini *et. al*. [50]. On the seventh day of culture, hippocampal slices were treated in the presence or absence of the NADPH inhibitors apocynin (100 μM, Sigma/Aldrich, St. Louis, MO) for 30 min, or diphenyleneiodomium (DPI 10 μM, Calbiochem, San Diego, CA) for 1 hr and the α7nAChR inhibitor methyllycaconitine (MLA 10 nM, Tocris, Ellisville, MO) for 30 min. Media was removed and replaced with media containing the inhibitors in the presence or absence of Aβ1-42 (100 nM; Calbiochem, San Diego, CA), or nicotine (500 nM; Sigma/Aldrich, St. Louis, MO) for 5 or 10 min, respectively.

Aβ preparation: Stock solution of synthetic rat Aβ1-42 (Calbiochem, San Diego, CA) was prepared at 100 mM in 200 mM HEPES, pH 8.0. The solution was gently agitated for ~ 30 min, aliquoted, and stored at -80°C. As previously published this method of Aβ preparation forms oligomers and not high molecular weight or fibrillar aggregate forms [3].

### Immunocytochemistry

After treatment with media in the presence or absence of the inhibitors and either Aβ (100 nM, 5 min) or nicotine (500 nM, 10 min) hippocampal slice cultures were fixed in 4% paraformaldehyde and 4% sucrose overnight at 4°C. Then the cultures were washed in phosphate buffer solution (PBS), permeabilized with 0.1% Triton X-100 and blocked with 4% normal goat serum for 30 min each. Slice cultures were incubated in phospho-ERK1/2 ([1:1000], Cell Signaling Technology Inc., Danvers, MA), NeuN (neuron-specific nuclear protein, [1:100], Millipore/Chemicon, Billerica, MA), MAP2 (microtubulin associated protein, [1:1000], Cell Signaling Technology Inc., Danvers, MA), GFAP (glial fibrillary acidic protein, [1:100], (Invitrogen/Molecular Probes, Carlsbad, CA), isolectin GS-IB4 (IB4, [1:1000], Invitrogen/Molecular Probes, Carlsbad, CA), and glutamic acid decarboxylase (GAD67, Millipore/Chemicon, Billerica, MA) overnight, rinsed with PBS and incubated for 2 hr with secondary antibodies Alexa 488 [1:200], and Alexa 594, [1:200], Invitrogen/Molecular Probes, Carlsbad, CA). Slices were washed in PBS, mounted on coverslips and analyzed using a Zeiss LSM 510 meta confocal microscope.

### Western blot hybridization

Hippocampal slices were homogenized in 10 mM HEPES, 1 mM EGTA, 1 mM EDTA, 150 mM NaCl, 50 mM NaF, and 10 mM Na_4_P_2_O_7 _supplemented with protease inhibitors, and phosphatase inhibitors (PI-I, PI-II, Sigma). The homogenate was sonicated and equivalent amounts of protein from each sample were resolved via 10% SDS-PAGE, transferred to Immobilon membranes, and incubated in I-Block (Applied Biosystems/Tropix, Foster City, CA). Blots then were incubated with primary antibodies phospho-ERK2 (1:3000), or ERK1/2 (1:3000) for 1 hr followed by horseradish peroxidase-linked secondary antibody and developed using enhanced chemiluminescence (Millipore/Amersham, Billerica, MA). Densitometric analyses of immunoreactivity were conducted using NIH image software.

### Statistical analysis

Statistical analyses were performed using a one-way ANOVA with Tukey's test for multiple comparisons to determine significant differences between control and treatment groups (control versus Aβ or nicotine; Aβ versus Aβ + drug; nicotine versus nicotine + drug). A *p *value of less than 0.05 was considered statistically significant. For Western blots analyses the results described represent at least n = 3 and each experimental condition contained replicates of between 6 - 8 slices.

## Competing interests

The authors declare that they have no competing interests.

## Authors' contributions

FS, performed experiments, analyzed data and wrote the manuscript. AC and CH helped with hippocampal cell culture preparation. RGP and JDS. helped with manuscript preparation. EK designed experiments and wrote the manuscript.
